# Impact of body mass index on postoperative oxygenation impairment in patients with acute aortic syndrome

**DOI:** 10.3389/fphys.2022.955702

**Published:** 2022-08-31

**Authors:** Chiyuan Zhang, Hui Bai, Yanfeng Zhang, Zhengyu Deng, Lei Zhang, Xuliang Chen, Zuli Fu, Ruizheng Shi, Guogang Zhang, Qian Xu, Guoqiang Lin

**Affiliations:** ^1^ Department of Cardiovascular Surgery, Xiangya Hospital, Central South University, Changsha, Hunan, China; ^2^ Department of Cardiovascular Medicine, The Third Xiangya Hospital, Central South University, Changsha, Hunan, China; ^3^ Department of Cardiovascular Medicine, Xiangya Hospital, Central South University, Changsha, Hunan, China

**Keywords:** body mass index, overweight, obesity, acute aortic syndrome, postoperative oxygenation impairment

## Abstract

**Objective:** Oxygenation impairment is a common complication of acute aortic syndrome (AAS) patients after surgical repair. The aim of this study is to identify the relationship between body mass index (BMI) and the risk of postoperative oxygenation impairment in AAS patients.

**Methods:** A total of 227 consecutive patients who were diagnosed as AAS and underwent surgical repair were recruited. They were divided into two groups based on the postoperative oxygenation impairment (non-oxygenation impairment group and oxygenation impairment group). Logistic regression was conducted to evaluate the association between BMI and the risk of oxygenation impairment after surgery. Dose-response curve and subgroup analysis were used to test the reliability of the results of regression analysis. A meta-analysis was then performed to further confirm these results using Pubmed, Embase, and Web of Science databases.

**Results:** For the retrospective study, a significant association was observed after adjusting for a series of variables. BMI was significantly correlated with postoperative oxygenation impairment in patients with AAS (OR, 95% CI, *P*: 1.27, 1.17–1.46, 0.001). Compared with the normal weight group (18.5 kg/m^2^ ≤ BMI <23.0 kg/m^2^), patients with excessive BMI were at a higher risk of oxygenation impairment for the overweight group (23.0 kg/m^2^ ≤ BMI <25 kg/m^2^) and obesity group (BMI ≥25 kg/m^2^) (OR, 95% CI, *P*: 4.96, 1.62–15.15, 0.005; 9.51, 3.06–29.57, <0.001). The dose-response curve showed that the risk of oxygenation impairment after surgery increased with the increased BMI. Besides, subgroup analysis showed that AAS patients who have an excess weight with a TNF-α ≥ 8.1 pg/ml carried an excess risk of postoperative oxygenation impairment. For the meta-analysis, the pooled result also indicated that AAS patients with high BMI had a significantly increased risk of oxygenation impairment after surgery (OR, 95% CI, *P*: 1.40, 1.18–1.66, 0.001).

**Conclusion:** Excessive BMI was an independent risk factor for AAS with postoperative oxygenation impairment.

## Introduction

Acute aortic syndrome (AAS) is a challenging medical emergency, which can be fatal and requires immediate surgical intervention. In recent years, despite increased advances in surgical techniques, the in-hospital mortality of the AAS remains above 25% due to a variety of postoperative complications (Trimarchi et al., 2005; Lee et al., 2018). One of the major complications is oxygenation impairment (Möller et al., 2021). Studies have shown that as many as 51% of AAS patients are complicated with oxygenation impairment after surgical repair (Nakajima et al., 2006). These patients often have a prolonged duration of mechanical ventilation, prolonged intensive care unit (ICU) stay, and increased incidence of hospital-acquired pneumonia, leading to higher hospital costs and poorer clinical prognosis (Zhao et al., 2021). Therefore, risk stratification is of great significance for postoperative oxygenation impairment in AAS patients.

Overweight and obesity are becoming relevant medical and socio-economic problems in contemporary society and have been recognized as among the most significant risk factors for cardiovascular diseases (Peitz et al., 2014; De Santo et al., 2018). Studies have revealed that an increased risk of postoperative oxygenation impairment occurred in acute aortic dissection (AD) patients with excessive body mass index (BMI) due to their disproportionately higher burden of comorbidities and underlying structural or functional changes (e.g., pulmonary vascular endothelial barrier disruption or dysfunction) in the lungs of these patients (Shen et al., 2018; Zhao et al., 2021). It is also reported that obese AD patients with postoperative oxygenation impairment exhibited a higher level of inflammation and oxidative stress, which contribute to the development of postoperative oxygenation impairment (Li et al., 2020). Moreover, excessive BMI is related to oxygenation impairment in ICU or postoperative patients (Filsoufi et al., 2008; De Jong et al., 2020). These studies demonstrate the potential value of excessive BMI for risk evaluation in AAS patients. However, other studies have shown that there is no association between excess weight and increased risk of oxygenation impairment, such as hypoxemia after Sun’s procedure (Wang KH et al., 2013; Gao et al., 2019), which makes the subject still questionable. Herein, we conducted a retrospective study to determine the relationship between BMI and postoperative oxygenation impairment in AAS patients. We then performed a meta-analysis to confirm or otherwise our findings. Our study aims to clarify these inconsistent findings, and provide new clinical evidence for risk stratification and further guidance in AAS patients with postoperative oxygenation impairment.

## Materials and methods

### Study population

After obtaining approval from the ethics committee of Xiangya Hospital (approval number 202101003), AAS patients who underwent surgery and were admitted to the Department of Cardiovascular Surgery at Xiangya Hospital from December 2018 to December 2020 were all retrospectively examined. The diagnosis was confirmed by aortic computed tomography angiography (CTA). The definition of AAS is AD with a patent false lumen and intramural hematoma (IMH) within 2 weeks of presentation. The exclusion criteria are listed as follows: 1) patients who had postoperative complications that may influence oxygenation indices including cardiogenic pulmonary edema, atelectasis, pneumothorax, and so on; 2) patients who only received medical therapy or refused surgical repair; 3) patients who died during operation or within 24 h after surgery; and 4) patients who had missing data more than 50% variables.

### Data collection and variables definition

The preoperative, intraoperative, and postoperative data were collected, including patients’ demography (age, gender, BMI, current smoking, etc.), vital signs (blood pressure, heart rate, etc.), comorbidities (hypertension, hyperlipemia, Marfan syndrome, etc.), perioperative laboratory tests and imaging examinations (white blood cell count, blood gas analysis, echocardiography etc.), operative details (operation time, cross-clamp time, plasma transfusion, etc.), and clinical outcomes (complications, mechanical ventilation time, ICU stay, etc.). Postoperative oxygenation impairment was defined as an arterial oxygen tension (P_aO2_)/inspiratory oxygen fraction (F_iO2_) ratio ≤200 with positive end-expiratory pressure (PEEP) ≥ 5 cm H_2_O at around 12 h (patients with a temporary, solvable conditions such as patient-ventilator conflict resulting in temporary hypo-oxygenation were reevaluated with another blood gas) after transferring patients to the ICU after surgery, which was also reported in other studies (Figueiredo et al., 2008; Ranieri et al., 2012; Yousefshahi et al., 2019). In addition, extubation ventilator was excluded from the study included that the patient was responsive and cooperative, P_aO2_ ≥ 80 mm Hg and F_iO2_ ≤ 0.4, breathing rate <30 times/min, and a stable hemodynamic state. All patients were divided into two groups according to the postoperative oxygenation impairment. In the stratified analysis, these patients were further subdivided into three groups based on the Asian criteria of BMI stratification: normal weight (18.5 kg/m^2^ ≤ BMI <23.0 kg/m^2^), overweight (23.0 kg/m^2^ ≤ BMI <25.0 kg/m^2^), and obesity (BMI ≥25 kg/m^2^) (Cui et al., 2018; Kim et al., 2021).

### Surgical procedure

All operations were performed by the same surgical team. The femoral artery or the axillary artery was selected as the site of cannulation. Briefly, for Stanford type A AAS, the aortic root procedure, entailing Bentall or David surgery was done according to the lesions of the aortic root. Besides, based on the length of the dissection, the ascending aorta, the ascending aorta with the proximal hemiarch, or the ascending aorta and the total arch were replaced or a stented elephant graft was released in the descending aorta. For most of the complicated Stanford type B AAS, thoracic endovascular aortic repair (TEVAR) was performed. Open surgical repair was performed in some situations, such as lower extremities artery disease, severe tortuosity of the iliac arteries, a sharp angulation of the aortic arch, and the absence of a proximal landing zone for the stent graft.

### Retrieval strategy, literature selection and quality assessment

The systematic literature search strategy followed the Preferred Reporting Items for Systematic Reviews and Meta-Analyses (PRISMA) guidelines (Liberati et al., 2009). The protocol was registered at the international prospective register of systematic reviews (PROSPERO) (ID number: CRD42022300844). Literature retrieval was conducted in PubMed, Embase, and Web of Science databases up to 1 January 2022. The main search terms were: acute aortic syndrome (including AD, IMH) AND postoperative AND oxygenation impairment (including acute respiratory distress syndrome, acute lung injury, hypoxemia) AND body mass index (including obesity).

Studies were included if they satisfied: 1) studies reported that the patients with AAS were diagnosed by contrast-enhanced computed tomography (CT) of the aorta and complicated with postoperative oxygenation impairment; 2) BMI was measured; 3) studies explored the relationship between BMI or excess weight (such as obesity) and oxygenation impairment after surgery; and 4) availability of an odds ratio (OR) or relative risk (RR) or hazard ratio (HR) and 95% Confidence Interval (CI). The exclusion criteria were: 1) articles of abstracts, letters, case reports, reviews, meta-analysis, and animal research type; 2) duplicated publications; and 3) articles with insufficient information. The clinical outcome of our analysis was limited to AAS with postoperative oxygenation impairment by any of these definitions.

### Data extraction

Two investigators (Chiyuan Zhang and Hui Bai) performed literature searches and reviewed the potential studies independently, and a third investigator (Yanfeng Zhang) was consulted to solve any disagreements. The following items from each study were recorded: first author’s surname, publication year, country, ethnicity, study size, sex, number of cases, diagnostic criteria of oxygenation impairment, BMI, OR/RR/HR value with the corresponding 95% CI and adjustment factors in the multivariable analysis. The quality of the included studies was assessed by the Newcastle-Ottawa Scale (NOS) score (Weis et al., 2019), and a score ≥6 was assigned as high-quality studies.

### Statistical analysis

In the retrospective analysis, data are reported as mean ± standard deviation for normally distributed continuous variables, as median and interquartile range (IQR) for non-normally distributed continuous variables, and as number and percentage for categorical variables. For quantitative data, Student *t* test or One-way ANOVA test were conducted for normally distributed values and Mann-Whitney *U* test was applied for non-normally distributed values. For qualitative data, Chi-squared test was performed for categorical values. Risk factors for the AAS with postoperative oxygenation impairment in association with the preoperative, intraoperative, and postoperative variables were analyzed by using univariate and multivariate logistic regression models, and a dose-response relationship curve was used to assess the relationship between BMI and postoperative oxygenation impairment in AAS patients. In addition, stratified analysis was conducted to evaluate different subgroups, including gender, age, Stanford classification, current smoking, CAD, hypertension, diabetes, and so on.

In the meta-analysis, the combined OR and 95% CI were used to identify the relationship between BMI and the risk of AAS with oxygenation impairment after surgery. Cochran’s *Q* test and *I* ^*2*^ statistics were utilized to evaluate the heterogeneity of studies included, and a random effect model was applied on the condition that *p* < 0.1 or *I* ^*2*^ ≥ 50%. Leave-one-out sensitivity analysis and subgroup analysis based on diagnostic criteria of oxygenation impairment, age, sample size, and different forms of BMI were further performed to explain the source of heterogeneity. Besides, funnel plot and Begg’s and Egger’s test were conducted to assess publication bias.

STATA 12.0 and R 4.0.3. Were used for statistical analysis and *p* < 0.05 (two-sided) was considered as statistical significance.

## Results

### Patients’ clinical characteristics

Overall, 245 patients with AAS were initially identified from the electronic healthcare records from Xiangya hospital. Among them, 18 patients were excluded owing to the postoperative cardiogenic pulmonary edema (n = 1), atelectasis (n = 3), pneumothorax (n = 2) and large pleural effusion (n = 2), death during operation or within 24 h after surgery (n = 6), and missing data for more than 50% variables (n = 4). Thus, a total of 227 patients with AAS were finally included in this present study (Supplementary Figure S1). The mean age of all patients was 52.19 ± 12.98 years, and 78.0% of them were male (177/227). The incidence of postoperative oxygenation impairment was 48.0% (n = 109), and patients were initially divided into two groups (the non-oxygenation impairment group and the oxygenation impairment group) based on it. Among the preoperative characteristics, male (OR, 95% CI, *P*: 2.1, 1.09–4.05, 0.026), BMI (OR, 95% CI, *P*: 1.24, 1.13–1.36, <0.001), obesity (OR, 95% CI, *P*: 4.42, 2.33–8.39, <0.001), hypertension (OR, 95% CI, *P*: 1.86, 1.04–3.33, 0.038), fatty liver (OR, 95% CI, *P*: 1.91, 1.02–3.60, <0.044), heart rate (OR, 95% CI, *P*: 1.02, 1.01–1.04, 0.010), hemoglobin (Hb) (OR, 95% CI, *P*: 1.02, 1.01–1.04, 0.004), and AD (OR, 95% CI, *P*: 7.58, 1.68–34.20, 0.008) were found to be significant risk factors for the occurrence of postoperative oxygenation impairment (Table 1). Among the intraoperative and postoperative characteristics, none of the intraoperative variables showed a predictive value (all *p* > 0.05) but postoperative mechanical ventilation time (OR, 95% CI, *P*: 1.01, 1.00–1.01, 0.032), pneumonia (OR, 95% CI, *P*: 1.83, 1.04–3.21, 0.036), and acute kidney injury (AKI) (OR, 95% CI, *P*: 2.56, 1.10–5.93, 0.029) were found to be related to AAS with postoperative oxygenation impairment (Table 2).

**TABLE 1:** The preoperative characteristics of patients with AAS.

	Non-oxygenation impairment (n = 118)	Oxygenation impairment (n = 109)	Or (95% CI)	*p* Values
Age (years)	53.00 (43.00–62.00)	52.00 (43.00–59.00)	1.00 (0.98–1.02)	0.629
Sex, male (%)	85 (72.0)	92 (84.4)	2.10 (1.09–4.05)	0.026
BMI (kg/m^2^)	23.88 ± 3.23	26.08 ± 3.15	1.24 (1.13–1.36)	<0.001
BMI (%)				
18.5 ≤ BMI<23 (n = 73)	53 (44.9)	20 (18.3)	References	—
23 ≤ BMI<25, (n = 42)	23 (19.5)	19 (17.4)	2.19 (0.99–4.85)	0.054
BMI ≥25, (n = 112)	42 (35.6)	70 (64.2)	4.42 (2.33–8.39)	<0.001
Current smoking, n (%)	59 (50.0)	59 (54.1)	1.18 (0.70–1.99)	0.534
Time from symptom onset to surgery (h)	25.39 (13.05–79.48)	20.67 (10.00–41.64)	1.00 (1.00–1.00)	0.308
Medical history				
Hypertension, n (%)	76 (64.4)	84 (77.1)	1.86 (1.04–3.33)	0.038
CAD, n (%)	12 (10.2)	8 (7.3)	0.70 (0.28–1.78)	0.454
Hyperlipemia, n (%)	5 (4.2)	3 (2.8)	0.64 (0.15–2.74)	0.547
Diabetes mellitus, n (%)	3 (2.5)	1 (0.9)	0.36 (0.04–3.46)	0.373
Fatty liver, n (%)	27 (31.0)	37 (46.3)	1.91 (1.02–3.60)	0.044
Marfan syndrome, n (%)	2 (1.7)	1 (0.9)	0.54 (0.05–6.01)	0.614
COPD, n (%)	2 (1.7)	2 (1.8)	1.08 (0.15–7.83)	0.936
Chronic liver disease, n (%)	3 (2.5)	6 (5.5)	2.23 (0.55–9.16)	0.265
Chronic kidney disease, n (%)	5 (4.2)	1 (0.9)	0.21 (0.02–1.82)	0.156
Preoperative oxygenation impairment, n (%)	29 (24.6)	31 (28.4)	1.22 (0.68–2.20)	0.510
Vital signs on admission				
Respiratory rate (/min)	20.00 (20.00–20.00)	20.00 (18.00–20.00)	1.02 (0.90–1.15)	0.768
SBP (mmHg)	140.32 ± 27.71	144.83 ± 33.60	1.01 (1.00–1.01)	0.270
DBP (mmHg)	69.00 (62.00–79.00)	74.00 (64.00–86.00)	1.01 (1.00–1.03)	0.082
Heart rate (/min)	74.00 (65.00–88.00)	82.00 (70.00–96.50)	1.02 (1.01–1.04)	0.010
Preoperative laboratory data				
Hb (g/L)	127.50 (113.00–136.00)	133.00 (122.50–145.00)	1.02 (1.01–1.04)	0.004
WBC (×10^9^/L)	9.85 (6.80–13.30)	11.10 (8.35–14.25)	1.03 (0.98–1.09)	0.279
PLT (×10^9^/L)	174.00 (135.50–241.25)	172.00 (118.00–212.50)	1.00 (0.99–1.00)	0.158
D-dimer (μg/ml)	1.52 (0.62–2.67)	1.40 (0.61–2.74)	0.98 (0.91–1.06)	0.624
FDP (mg/L)	15.50 (5.95–32.83)	13.55 (6.95–29.30)	1.00 (0.99–1.01)	0.704
Cr (μmol/L)	88.05 (72.58–108,30)	96.40 (82.55–116.15)	1.00 (1.00–1.00)	0.402
CRP (mg/L)	51.25 (7.39–90.08)	35.20 (9.15–107.70)	1.00 (0.99–1.01)	0.992
Preoperative ultrasound and CT findings				
Stanford classification				
Type A, n (%)	79 (67.5)	78 (71.6)	References	—
Type B, n (%)	38 (32.5)	31 (28.4)	0.83 (0.47–1.46)	0.510
Aortic dissection, n (%)	97 (87.4)	105 (98.1)	7.58 (1.68–34.20)	0.008
Intramural hematoma, n (%)	21 (17.8)	14 (13.1)	0.65 (0.31–1.35)	0.243
LVEDD (mm)	50.00 (45.00–55.00)	51.00 (47.00–54.00)	1.00 (0.96–1.04)	0.901
LVEF (%)	60.00 (56.00–66.00)	60.00 (56.00–65.00)	0.98 (0.95–1.02)	0.337
Aortic regurgitation, n (%)	92 (84.4)	84 (84.8)	1.04 (0.49–2.20)	0.929
Pleural effusion, n (%)	39 (40.2)	40 (43.5)	1.14 (0.64–2.04)	0.649
Medications on admission				
Vasodilators, n (%)	73 (61.9)	80 (73.4)	1.70 (0.97–3.00)	0.065

Data are presented as mean ± SD, n (%), or medians (interquartile ranges). *p* values for univariate logistic regression. AAS, acute aortic syndrome; OR, odds ratio; CI, confidence interval; BMI, body mass index; CAD, coronary artery disease; COPD, chronic obstructive pulmonary disease; SBP, systolic blood pressure; DBP, diastolic blood pressure; WBC, white blood cell; Hb, hemoglobin; PLT, platelet; FDP, fibrinogen degradation products; Cr, creatinine; CRP, C reactive protein; LVEDD, left ventricular end-diastolic dimension; LVEF, left ventricular ejection fraction.

**TABLE 2:** The intraoperative and postoperative characteristics of patients with AAS.

	Non-oxygenation impairment (n = 118)	Oxygenation impairment (n = 109)	OR (95% CI)	*p* Values
Intraoperative data				
Aortic root replacement, n (%)	65 (55.1)	69 (63.3)	1.41 (0.83–2.40)	0.209
Hemiarch replacement, n (%)	9 (7.6)	10 (9.2)	1.22 (0.48–3.13)	0.674
Total arch replacement, n (%)	61 (51.7)	67 (61.5)	1.49 (0.88–2.53)	0.139
Stented elephant trunk, n (%)	58 (49.2)	64 (58.7)	1.47 (0.87–2.49)	0.149
Bentall operation, n (%)	23 (19.5)	14 (12.8)	0.61 (0.30–1.25)	0.178
David operation, n (%)	2 (1.7)	0 (0.0)	—	—
TEVAR, n (%)	34 (28.8)	26 (23.9)	0.77 (0.43–1.40)	0.398
CABG operation, n (%)	6 (5.1)	3 (2.8)	0.53 (0.13–2.17)	0.375
CPB time (h)	3.43 (2.82–4.07)	3.43 (2.77–4.39)	1.04 (0.87–1.24)	0.694
Cross-clamp time (h)	1.64 (1.22–2.44)	1.52 (1.21–2.08)	0.95 (0.71–1.28)	0.751
Circulatory arrest time (min)	30.00 (21.00–36.00)	30.00 (22.00–40.00)	1.00 (0.98–1.01)	0.538
Operation time (h)	6.33 (2.04–7.69)	6.75 (4.92–8.42)	1.07 (0.99–1.15)	0.082
Nasopharyngeal temperature (°C)	25.20 (24.35–26.50)	25.00 (24.30–27.00)	0.98 (0.89–1.07)	0.604
RBC transfusion (u)	4.00 (2.00–6.00)	4.00 (2.00–5.50)	1.01 (0.95–1.09)	0.680
PLT transfusion (u)	1.00 (1.00–2.00)	1.00 (1.00–2.00)	1.00 (0.99–1.01)	0.703
Plasma transfusion (ml)	550.00 (350.00–650.00)	500.00 (380.00–600.00)	1.00 (1.00–1.00)	0.755
Postoperative data				
CRP (mg/L)	136.00 (85.05–176.25)	124.29 (78.20–199.00)	1.00 (1.00–1.00)	0.885
Mechanical ventilation time (h)	37.40 (16.47–81.22)	89.61 (36.45–138.38)	1.01 (1.00–1.01)	0.032
Tracheotomy, n (%)	113 (95.8)	109 (100.0)	4.78 (0.55–41.57)	0.156
Pneumonia, n (%)	71 (60.2)	80 (73.4)	1.83 (1.04–3.21)	0.036
Cerebral infarction, n (%)	5 (4.2)	6 (5.5)	1.32 (0.39–4.44)	0.658
Delirium, n (%)	39 (33.1)	44 (40.4)	1.37 (0.80–2.36)	0.253
AKI, n (%)	9 (7.6)	19 (17.4)	2.56 (1.10–5.93)	0.029
Renal replacement therapy, n (%)	6 (5.1)	8 (7.3)	1.48 (0.50–4.41)	0.483
ICU stay (days)	5.00 (3.75–8.00)	6.00 (4.00–9.00)	1.01 (0.96–1.05)	0.762
Hospital stay (days)	12.00 (9.00–18.00)	13.00 (9.50–18.00)	1.00 (0.97–1.03)	0.913
In hospital death, n (%)	7 (5.9)	12 (11.0)	1.96 (0.74–5.18)	0.174

Data are presented as mean ± SD, n (%), or medians (interquartile ranges). *p* values for univariate logistic regression. AAS, acute aortic syndrome; OR, odds ratio; CI, confidence interval; TEVAR, thoracic endovascular aortic repair; CABG, coronary artery bypass grafting; CPB, cardiopulmonary bypass; RBC, red blood cell; PLT, platelet; CRP, C reactive protein; AKI, acute kidney injury; ICU, intensive care unit.

Stratified analysis was then conducted according to BMI, which is presented in Supplementary Table S1 and Supplementary Table S2. The preoperative data showed that a fatty liver, preoperative oxygenation impairment, and a higher white blood cell (WBC) were more common in the obesity group (all *p* < 0.05), and the proportion of male and hypertension, and diastolic blood pressure (DBP) were elevated in the excessive BMI group (both overweight and obesity group) (all *p* < 0.05) (Supplementary Table S1). Besides, the intraoperative and postoperative data indicated that mechanical ventilation time was increased in these groups (*p* < 0.05), and the proportion of Bentall and coronary artery bypass grafting (CABG) operation had a negative correlation with BMI, while postoperative oxygenation impairment has a positive correlation with BMI (all *p* < 0.05) (Supplementary Table S2).

### The association between BMI and AAS with postoperative oxygenation impairment

**FIGURE 1:**
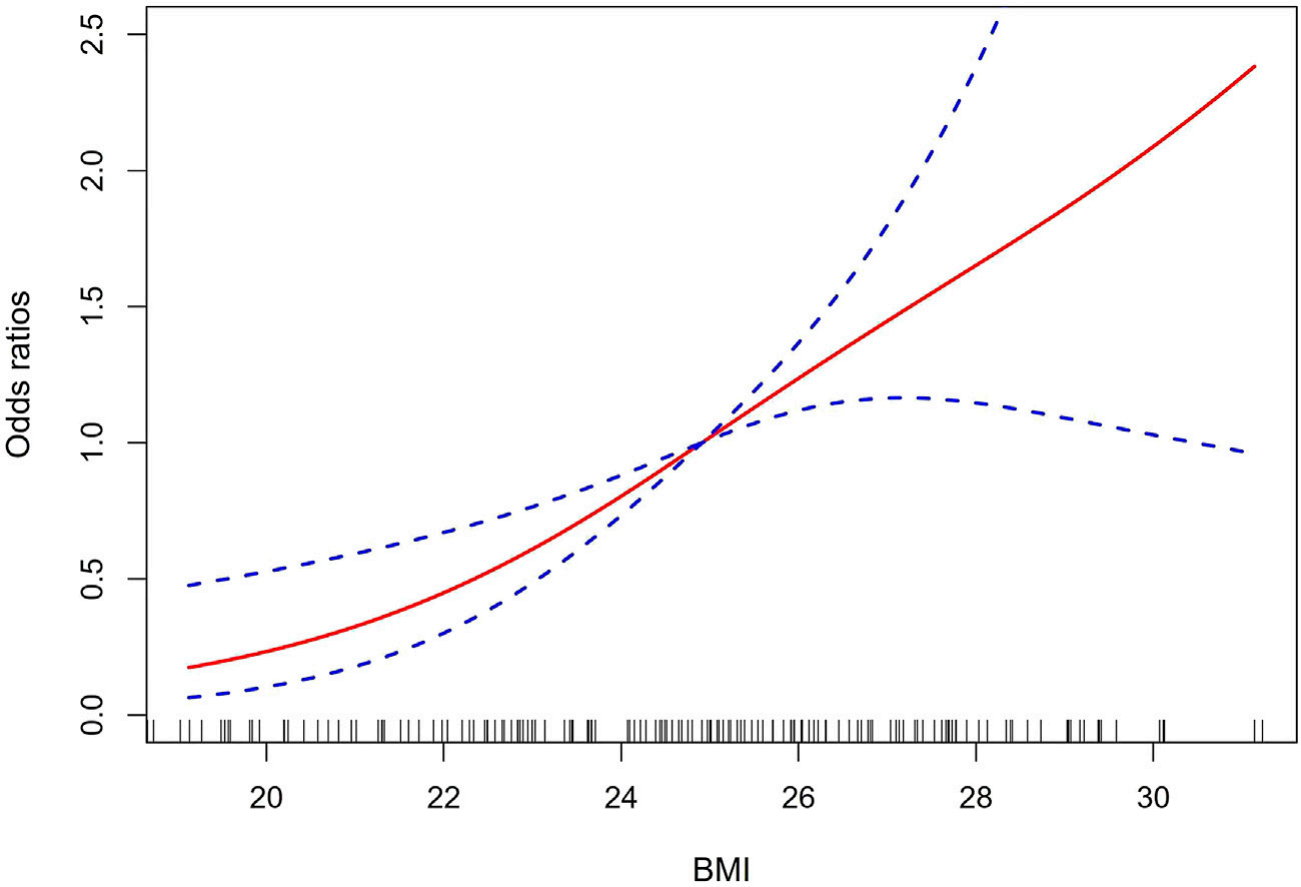
The dose-response relationship analysis of BMI and AAS with postoperative oxygenation impairment. The solid red line and the dashed-blue line represent the estimated OR and 95% CI, respectively. BMI, body mass index; AAS, acute aortic syndrome; OR, odds ratio; CI, confidence interval.

To determine the relationship between BMI and AAS with postoperative oxygenation impairment, a multivariate logistic regression analysis was further performed. After adjusting to sex, hypertension, fatty liver, hyperlipemia, diabetes mellitus, heart rate, Hb, preoperative oxygenation impairment, aortic dissection, mechanical ventilation time, pneumonia, and AKI, BMI remained significantly correlated with postoperative oxygenation impairment in patients with AAS (OR, 95% CI, *P*: 1.27, 1–1.46, 0.001) and excess weight. Both overweight and obesity were independent risk factors (OR, 95% CI, *P*: 4.96, 1.62–15.15, 0.005; 9.51, 3.06–29.57, <0.001), which are presented in Table 3. Our study then explored the non-linear correlation between BMI and AAS with postoperative oxygenation impairment. The dose-response relationship analysis suggests that the risk of oxygenation impairment after surgery increased with the increased BMI, which is presented in Figure 1. To determine the development of its effect sizes, interaction analysis was also performed and all patients were divided into different subgroups according to sex, age, current drinking, coronary artery disease (CAD), chronic obstructive pulmonary disease (COPD), systolic blood pressure (SBP), preoperative WBC, and so on. As shown in Table 4, there was no interaction in most strata (*P* for interaction = 0.057–0.904), and only AAS patients with excessive BMI who had a tumor necrosis factor-α (TNF-α) ≥ 8.1 pg/ml had an excess risk of postoperative oxygenation impairment (*p* = 0.027).

**TABLE 3:** Multivariate logistic regression analysis for AAS with postoperative oxygenation impairment.

	OR (95% CI)	*p* Values
Sex, male (%)	1.14 (0.41–3.16)	0.809
BMI (kg/m^2^)	1.27 (1.10–1.46)	0.00
BMI (n, %)		
18.5 ≤ BMI< 23	References	—
23 ≤ BMI <25	4.96 (1.62–15.15)	0.005
BMI ≥25	9.51 (3.06–29.57)	<0.001
Hypertension, n (%)	0.63 (0.27–1.44)	0.271
Hyperlipemia, n (%)	0.18 (0.03–1.35)	0.096
Diabetes mellitus, n (%)	0.56 (0.02–15.44)	0.730
Fatty liver, n (%)	1.18 (0.48–2.88)	0.725
Preoperative oxygenation impairment, n (%)	0.52 (0.22–1.28)	0.155
Heart rate (/min)	1.01 (0.99–1.03)	0.475
Hb (g/L)	1.02 (1.00–1.04)	0.055
Aortic dissection, n (%)	11.62 (1.07–126.55)	0.052
Mechanical ventilation time (h)	1.00 (1.00–1.00)	0.605
Pneumonia, n (%)	1.54 (0.68–3.47)	0.302
AKI, n (%)	2.13 (0.69–6.56)	0.187

Adjusted to sex, hypertension, fatty liver, hyperlipemia, diabetes mellitus, heart rate, Hb, preoperative oxygenation impairment, aortic dissection, mechanical ventilation time, pneumonia and AKI. AAS, acute aortic syndrome; OR, odds ratio; CI, confidence interval; BMI, body mass index; Hb, hemoglobin; AKI, acute kidney injury.

**TABLE 4:** Subgroup analysis and interaction analysis of the relationship between BMI and AAS with postoperative oxygenation impairment.

	No. of patients	BMI levels (kg/m^2^)			*P* For interaction
		<23	23–25	≥25	
Sex					0.482
Male	177	ref	3.86 (1.51–9.86)	4.30 (1.96–9.44)	
Female	50	ref	1.50 (0.22–10.36)	2.00 (0.55–7.24)	
Age, years					0.500
<60	165	ref	4.63 (1.72–12.47)	4.63 (1.99–10.75)	
≥60	62	ref	1.43 (0.27–7.44)	2.56 (0.84–7.83)	
Stanford classification					0.743
Type A	157	ref	3.96 (1.46–10.75)	3.68 (1.68–8.06)	
Type B	69	ref	2.40 (0.57–10.04)	3.84 (1.14–12.95)	
AD					0.539
No	16	ref	—	3.33 (0.36–30.70)	
Yes	202	ref	3.39 (1.43–8.05)	4.12 (2.02–8.39)	
IMH					0.326
No	183	ref	3.30 (1.32–8.27)	4.59 (2.15–9.79)	
Yes	35	ref	8.75 (0.74–103.82)	2.19 (0.47–10.21)	
Current smoking					0.198
No	109	ref	2.45 (0.84–7.21)	2.05 (0.85–4.90)	
Yes	118	ref	6.14 (1.58–23.79)	8.61 (2.71–27.39)	
Current drinking					0.274
No	171	ref	4.48 (1.76–11.43)	3.52 (1.68–7.38)	
Yes	56	ref	1.33 (0.22–7.98)	3.62 (0.81–16.15)	
Fatty liver					0.500
No	103	ref	3.90 (1.34–11.34)	4.80 (1.70–13.59)	
Yes	64	ref	15.00 (1.03–218.30)	6.91 (0.75–63.53)	
Hypertension					0.884
No	67	ref	2.25 (0.44–11.48)	3.41 (1.07–10.86)	
Yes	160	ref	3.25 (1.18–8.96)	3.37 (1.42–7.99)	
CAD					0.533
No	207	ref	3.10 (1.34–7.14)	3.25 (1.65–6.38)	
Yes	20	ref	—	—	
Diabetes					0.564
No	223	ref	3.24 (1.42–7.38)	3.54 (1.83–6.85)	
Yes	4	ref	—	—	
COPD					0.261
No	223	ref	3.61 (1.58–8.26)	3.90 (2.00–7.62)	
Yes	4	ref	—	—	
SBP, mmHg					0.057
<140	104	ref	4.14 (1.35–12.66)	2.07 (0.83–5.17)	
≥140	123	ref	2.69 (0.76–9.59)	6.55 (2.41–17.83)	
DBP, mmHg					0.658
<90	192	ref	3.77 (1.57–9.05)	3.65 (1.78–7.51)	
≥90	35	ref	1.33 (0.14–12.82)	3.25 (0.63–16.79)	
Preoperative WBC, 10^9^/L					0.209
<9.5	93	ref	1.89 (0.59–6.07)	1.89 (0.72–5.00)	
≥9.5	134	ref	5.57 (1.74–17.86)	5.95 (2.31–15.27)	
Preoperative D-dimer, μg/mL					0.385
<0.5	42	ref	1.14 (0.15–8.59)	2.55 (0.52–12.37)	
≥0.5	184	ref	4.27 (1.71–10.66)	3.99 (1.93–8.25)	
Preoperative FDP, mg/L					0.904
<5	36	ref	3.00 (0.42–21.30)	3.00 (0.58–15.61)	
≥5	184	ref	3.50 (1.41–8.68)_	4.02 (1.91–8.44)	
Preoperative Cr, μmol/L					0.349
<111	168	ref	2.54 (0.96–6.70)	4.01 (1.89–8.52)	
≥111	59	ref	5.40 (1.12–26.04)	2.80 (0.73–10.76)	
Preoperative P_aO2_/F_iO2_ ratio					0.592
≤200	60	ref	2.46 (1.27–6.63)	5.32 (1.75–8.74)	
>200	167	ref	2.19 (0.95–5.06)	4.78 (2.28–10.02)	
Preoperative ESR, mm/h					0.616
<20	65	ref	7.71 (1.25–47.75)	4.91 (1.57–15.31)	
≥20	80	ref	3.21 (0.83–12.47)	3.12 (1.03–9.46)	
Preoperative CRP, mg/l					0.272
<8	23	ref	1.13 (0.08–16.31)	1.69 (0.25–11.34)	
≥8	70	ref	12.80 (2.02–81.12)	10.35 (2.09–51.30)	
Preoperative IL-1β, pg/ml					0.672
<5.0	43	ref	2.89 (0.33–25.70)	3.00 (0.66–13.66)	
≥5.0	50	ref	4.50 (0.82–24.57)	6.00 (1.46–24.69)	
Preoperative TNF-α, pg/ml					0.788
<8.1	34	ref	4.67 (0.53–40.89)	2.80 (0.45–17.38)	
≥8.1	49	ref	4.53 (0.75–27.39)	5.10 (1.34–19.47)	
Preoperative IL-6, pg/ml					0.078
<5.0	3	ref	—	—	
≥5.0	90	ref	3.57 (0.93–13.66)	4.70 (1.68–13.15)	
Preoperative IL-10, pg/ml					0.763
<9.1	58	ref	5.63 (1.02–30.90)	4.18 (1.12–15.65)	
≥9.1	34	ref	2.00 (0.24–16.61)	3.43 (0.65–18.22)	
Aortic regurgitation					0.349
No	32	ref	6.00 (0.58–61.82)	5.20 (0.71–37.90)	
Yes	176	ref	1.02 (0.42–2.48)	1.77 (0.90–3.44)	
Pleural effusion					0.373
No	110	ref	2.92 (0.83–10.27)	4.33 (1.69–11.08)	
Yes	79	ref	3.82 (1.05–13.91)	2.06 (0.69–6.15)	
Postoperative ESR, mm/h					0.511
<20	15	ref	2.50 (0.10–62.51)	1.25 (0.12–13.24)	
≥20	69	ref	6.25 (1.15–34.12)	6.25 (1.78–22.01)	
Postoperative CRP, mg/l					—
<8	1	ref	—	—	
≥8	77	ref	5.25 (1.13–24.42)	4.09 (1.18–14.15)	
Postoperative IL-1β, pg/ml					0.072
<5.0	33	ref	1.00 (0.13–8.00)	1.33 (0.27–6.50)	
≥5.0	37	ref	—	6.67 (1.16–38.25)	
Postoperative TNF-α, pg/ml					0.027
<8.1	14	ref	0.67 (0.03–18.06)	0.11 (0.01–1.78)	
≥8.1	50	ref	12.50 (1.60–97.65)	6.36 (1.46–27.67)	
Postoperative IL-6, pg/ml					—
<5.0	0	ref	—	—	
≥5.0	70	ref	4.50 (0.94–21.56)	3.00 (0.97–9.30)	
Postoperative IL-10, pg/ml					0.658
<5.9	14	ref	6.00 (0.22–162.53)	1.50 (0.09–25.39)	
≥5.9	56	ref	3.20 (0.48–21.17)	3.43 (0.99–11.85)	

BMI, body mass index; AAS, acute aortic syndrome; CAD, coronary artery disease; COPD, chronic obstructive pulmonary disease; AD, aortic dissection; IMH, intramural hematoma; SBP, systolic blood pressure; DBP, diastolic blood pressure; WBC, white blood cell; FDP, fibrinogen degradation products; Cr, creatinine; ESR, erythrocyte sedimentation rate; CRP, C reactive protein; IL-1β, Interleukin-1β; TNF-α, Tumor necrosis factor-α; IL-6, Interleukin-6; IL-10, Interleukin-10.

**FIGURE 2:**
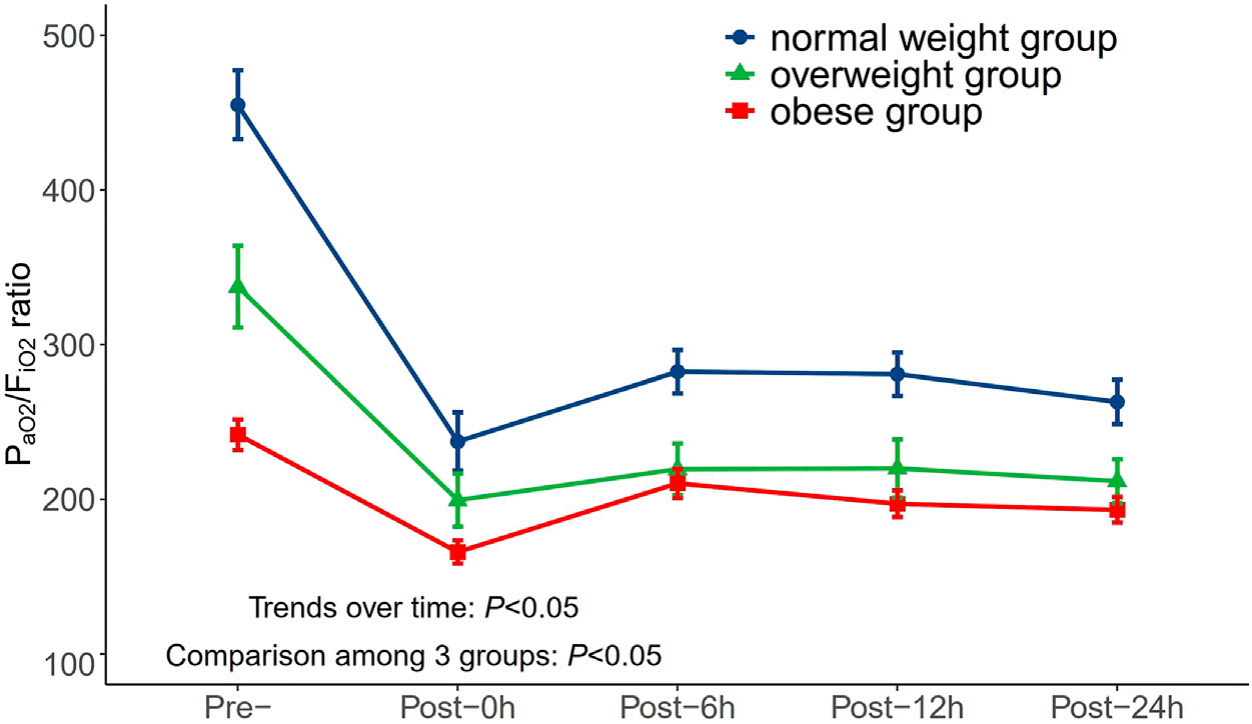
Changes of P_aO2_/F_iO2_ ratio in different BMI groups over time during perioperative period. P_aO2_/F_iO2_, arterial oxygen tension/inspiratory oxygen fraction; BMI, body mass index.

Moreover, the mean P_aO2_/F_iO2_ ratio was observed in the normal weight, overweight, and obesity groups during the perioperative period (Figure 2). Figure 2 indicates that AAS patients with increased BMI values were related to a lower P_aO2_/F_iO2_ ratio on admission and at 0, 6, 12, and 24 h after surgery (*p* < 0.05). These trends did not change over time (*p* < 0.05).

### Literature search results and studies’ characteristics of meta-analysis

Based on this retrieval strategy, 66 articles were initially identified, of which 23 were excluded due to duplication. A list of 14 articles was then selected for further full-text review after screening the titles or abstracts. Finally, eight articles were included in the quantitative analysis by removing six articles due to the lack of OR value or other necessary data. The literature screening flow diagram is shown in Supplementary Figure S2.

All of the studies were published between 2006 and 2021, and their characteristics are summarized in Supplementary Table S3. In detail, these studies were case-control studies involving a sum of 1,451 patients with AAS. They were all conducted in China, apart from one study performed in Japan. Six studies defined postoperative oxygenation impairment as P_aO2_/F_iO2_ ratio ≤200, while two studies used P_aO2_/F_iO2_ ratio ≤100. Besides, according to the NOS criteria, the studies included were of high quality with scores ranging from seven to eight, which is shown in Supplementary Figure S3.

### Quantitative synthesis and analysis of meta-analysis

**FIGURE 3:**
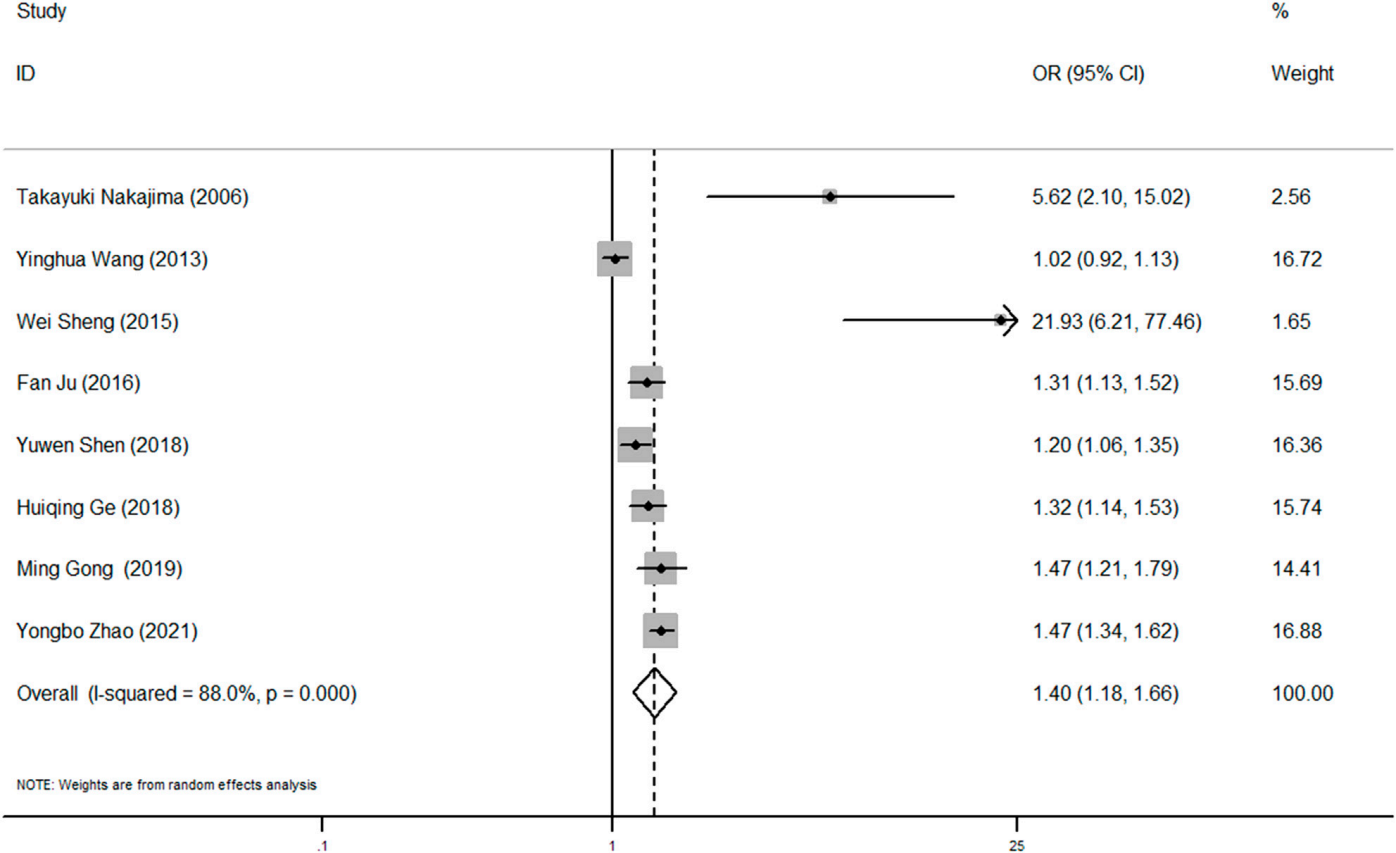
Forest plot of OR of BMI and postoperative oxygenation impairment in AAS patients. OR, odds ratio; BMI, body mass index; AAS, acute aortic syndrome.

The pooled result revealed that AAS patients with high BMI had a significantly increased risk of postoperative oxygenation impairment (OR, 95% CI, *P*: 1.40, 1.18–1.66, 0.001) and significant heterogeneity was found among these studies (*I* ^*2*^ = 88.0%, *p* < 0.001) (Figure 3).

To identify the potential source of heterogeneity in these studies, subgroup and sensitivity analyses were conducted. As shown in Supplementary Table S4, the pooled OR values were higher in studies with P_aO2_/F_iO2_ ratio ≤100 as diagnostic criteria of postoperative oxygenation impairment, age >50 years, sample size ≤200 or BMI as categorical data. The subgroup results also suggested that there was a consistently significant association between high BMI and increased risk of oxygenation impairment. In addition, the leave-one-out sensitivity analysis indicated that no study had a large significant effect on the main results (Supplementary Figure S4). For the evaluation of publication bias, both Begg’s and Egger’s test were not significant (*p* = 0.108, 0.060). However, the funnel plot was basically asymmetrical, which remained a potential publication bias (Supplementary Figure S5).

## Discussion

In this retrospective study, we enrolled 227 AAS patients and found excess weight to be an independent risk factor of AAS with postoperative oxygenation impairment. Besides, the results of the meta-analysis further confirmed the positive correlation between BMI and the increased risk of oxygenation impairment after surgery in patients with AAS.

AAS often requires emergency surgery owing to its urgent onset, rapid progression, and high mortality. Postoperative oxygenation impairment is one of the most common complications after surgery, and it has an adverse impact on the duration of mechanical ventilation and the length of ICU stay (Liu et al., 2017). Therefore, assessing the risk factors predisposing to this complication is crucial for clinical outcomes. With the increasing incidence of overweight and obese patients, studies have increasingly focused on the relationship between overweight and obesity, and AD patients who have postoperative oxygenation impairment; however, the results are controversial. Shen et al. (2018) performed a retrospective analysis of 169 consecutive patients with Stanford type A AD who underwent a total arch replacement procedure and found that excessive BMI increased the risk of postoperative oxygenation impairment. In addition, Nakajima et al. (2006) demonstrated that BMI ≥25 kg/m^2^ was an independent risk factor of hypoxemia in acute AD patients after surgery. However, Wang Y et al. (2013) revealed that BMI was related to postoperative hypoxemia in Stanford type A AD patients but not independently after adjusting for clinical variables in multivariate analysis. Similarly, another two studies from China indicated that there was no significant correlation between BMI or excess weight and postoperative hypoxemia following surgical repair of AAS (Liu et al., 2017; Gao et al., 2019). These inconsistent findings are likely to be due to differences in diagnostic criteria for oxygenation impairment, heterogeneity in the study population, and different variables adjustment for risk stratification.

In the present study, we included 227 patients with AAS who underwent surgical treatment and found that the rate of oxygenation impairment after surgery was 48.0%, which is in line with the previous studies (Shen et al., 2018). We stratified all patients based on BMI into three groups (normal weight, overweight, and obese), and a significant positive correlation between BMI and postoperative oxygenation impairment was determined. A multivariate logistic regression analysis showed that excess weight (both overweight and obesity) was an independent risk factor for oxygenation impairment. A dose-response relationship curve and subgroup analysis further confirmed this conclusion. We also compared the mean P_aO2_/F_iO2_ ratio among three groups at different perioperative timepoints and found that the group with a higher BMI had a lower P_aO2_/F_iO2_ ratio. The variance trend analysis showed no change over time. Moreover, we reviewed eight studies for meta-analysis. Based on the NOS criteria, the included studies were of high quality with a score of seven or eight, which suggests that the results are reliable. The pooled results support the results of our retrospective study and indicate that excessive BMI increased the risk of postoperative oxygenation impairment in AAS patients.

To date, the mechanism of postoperative oxygenation impairment in AAS has not been fully elucidated; however, it is currently believed that inflammation plays an important role in this process (Zhou et al., 2021). Several inflammatory reactants can be used as clinical biomarkers. For instance, Gong et al. (2019) reported that Stanford type A AD patients with postoperative oxygenation impairment exhibited a high level of preoperative WBC. Meanwhile, Ge et al.bookmark://bib_Ge_et_al_2018/ (2018) provided further confirmation in their prediction model for postoperative hypoxemia. In addition, increased serum C-reactive protein and D-dimer level were associated with hypoxia after surgery in AD patients, which provided potential clinical biomarkers (Liao et al., 2021). Several studies have shown that some surgical factors (e.g., cardiopulmonary bypass, circulatory arrest, plasma transfusion, etc.) can enlarge the local vascular inflammation and increase the release of inflammatory factors into circulation. Furthermore, augmentative systemic inflammation leads to lung tissue damage and dysfunction in AD patients (Ju et al., 2016; Shen et al., 2018). However, few studies have reported its pathogenesis in obese AAS patients. It is reported that obese AAS patients exhibited an elevated level of Interleukin-1β (IL-1β), Tumor necrosis factor-α (TNF-α) and Interleukin-6 (IL-6) (Wu et al., 2020), while our study has also shown that these patients had a higher preoperative WBC and preoperative and postoperative C reactive protein (CRP) (*p* = 0.034, 0.253, 0.455), which suggests that they have a more intense level of inflammation and oxidative stress. These inflammatory reactants could be produced by adipose tissue and under the surgical stress can further develop into systemic inflammation through circulation, destroy the pulmonary capillary bed, and cause pulmonary interstitial edema, leading to mismatched ventilation/perfusion distribution and oxygenation impairment (Wu et al., 2016).

AAS patients with high BMI often suggest endocrine disorders and they are environmental exposed daily to several endocrine disrupters at very low doses, which are strictly related to several cardiovascular risk factors including obesity, metabolic disorders, and diabetes (Wang Y et al., 2013; Quagliariello et al., 2019). For instance, Bisphenol A (BPA) is an endocrine disrupter that is commonly used in the manufacturing of plastics and has been shown to stimulate the intracellular calcium accumulation and lipid peroxidation, and increase the production of interleukins, causing pro-oxidative and pro-inflammatory effects on the cardiovascular system (Quagliariello et al., 2019). As a result, these environmental factors can magnify the inflammatory response and expand tissue damage in these patients. Furthermore, excessive BMI adversely affects the physiology of the respiratory system and excess adipose tissue can impair respiratory compliance, increase the respiratory load, and reduce ventilatory drive, which has been treated as a risk factor for hypoxemia after cardiac surgery (Weiss et al., 2000; Santos et al., 2013). The high burden of comorbidities such as subclinical renal injury in overweight or obese patients could be another reason for the increased incidence of postoperative oxygenation impairment (Shi et al., 2020).

Our subgroup analysis revealed that there was no interaction in most strata, confirming the reliability of excessive BMI as an independent risk factor for postoperative oxygenation impairment in AAS patients. However, it has also shown that AAS patients with a high BMI and a TNF-α level ≥8.1 pg/ml had an excess risk of oxygenation impairment after surgery. The reason for this result may be that TNF-α, as an inflammatory factor, participates in the occurrence and development of AAS with postoperative oxygenation impairment, which was mentioned earlier (Wu et al., 2020).

It is believed that excessive BMI has a negative impact on respiratory function after a major cardiac surgery, such as coronary artery bypass grafting (CABG) (Apostolakis et al., 2010; Ranucci et al., 2014). However, there are few reports on the effect of BMI on AAS with postoperative oxygenation impairment, and its role still needs to be confirmed by further studies. Our study focused on patients with AAS, and for the first time explored the relationship between BMI and postoperative oxygenation impairment in them, which has provided potent evidence for risk stratification and further clinical management in such patients. For them, protective intraoperative mechanical ventilation (e.g., an intraoperative high level of PEEP and alveolar recruitment maneuvers) is necessary (Bluth et al., 2019). Besides, nitric oxide inhalation therapy after operation (5–10 ppm, over 24 h) may attenuate postoperative hypoxemia, which has been reported in obese patients with acute type A aortic dissection (Zheng et al., 2022). Due to the important role of inflammation in its pathogenesis, some studies have also shown that perioperative anti-inflammatory therapy, including high-dose ulinastatin (20,000 units/kg), can reduce lung injury and improve the respiratory function by attenuating the elevation of cytokines and polymorphonuclear neutrophil elastase (PMNE) (Xu et al., 2013; Duan et al., 2018). These measures may reduce the risk of AAS with postoperative oxygenation impairment. However, further large-scale research is needed to confirm their efficacy. Finally, proper nutrition and nutraceuticals are also important long-term treatment for these patients (Raynor and Champagne, 2016; Berretta et al., 2020). On the one hand, proper nutrition (e.g., the increased consumption of fruits and vegetables) can promote patients to achieve a state of negative energy balance, which is a key part of weight management (Raynor and Champagne, 2016). On the other hand, some nutraceuticals, particularly ascorbic acid (ASC), by affecting neutrophil migration and apoptosis and stimulating phagocytosis, exert an antioxidant and immunomodulatory effect, which show a good prospect in improving postoperative inflammation in AAS. It is also reported that oral administration of ASC can reduce arterial stiffness and enhance endothelial function (Berretta et al., 2020).

This study has observed some limitations. First, due to its retrospective nature and relatively small sample size, the results in our study may not be universally representative. Second, it is difficult to assess the impact of surgical procedures on the AAS with postoperative oxygenation impairment owing to differences in surgical details. Third, our meta-analysis included case-control studies and significant heterogeneity might have increased the bias of the results. Further prospective studies and an in-depth mechanism exploration will be needed to examine the results of this study.

## Conclusion

Our findings show that high BMI patients with AAS are more likely to have oxygenation impairment after surgery and excessive BMI is an independent risk factor for AAS with postoperative oxygenation impairment.

## Data Availability

The original contributions presented in the study are included in the article/Supplementary Material, further inquiries can be directed to the corresponding authors.
