# Supplementary material for: Impact of body mass index on postoperative oxygenation impairment in patients with acute aortic syndrome
Source: Front Physiol. 2022 Aug 31;13:955702. doi: 10.3389/fphys.2022.955702 (PMC9470752; doi:10.3389/fphys.2022.955702)
Supplement: Supplementary file 1 [file Table1.DOCX]

**Supplementary Figure 1**


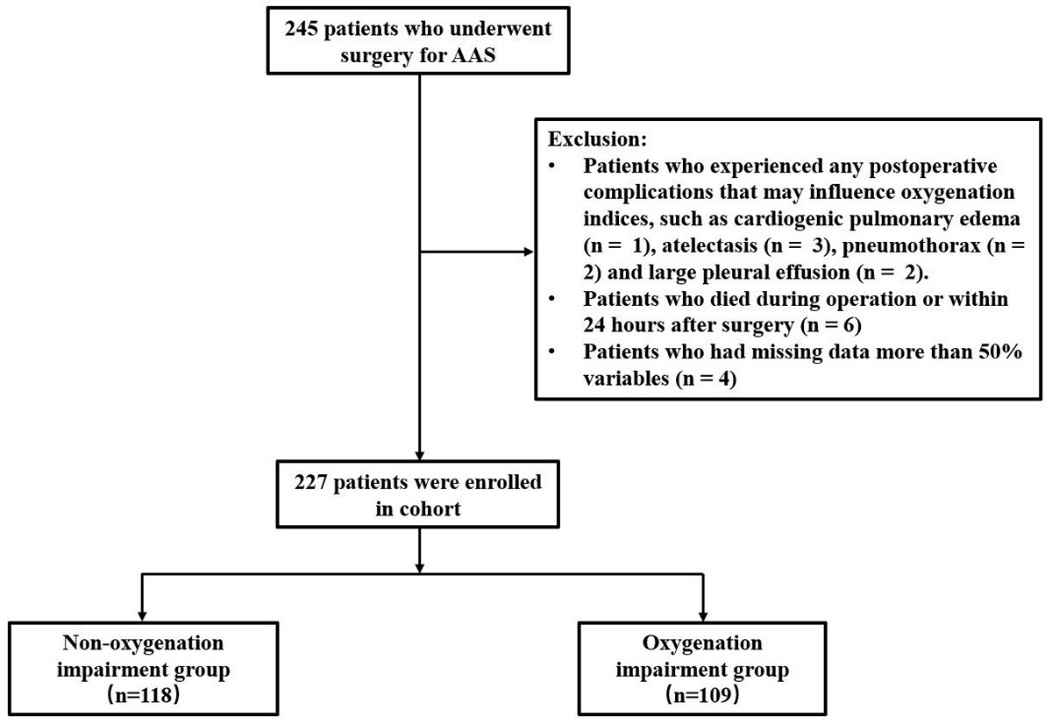


**Supplementary Figure 1.** Study flow chart in the retrospective study. AAS: acute aortic syndrome.

**Supplementary Figure 2**


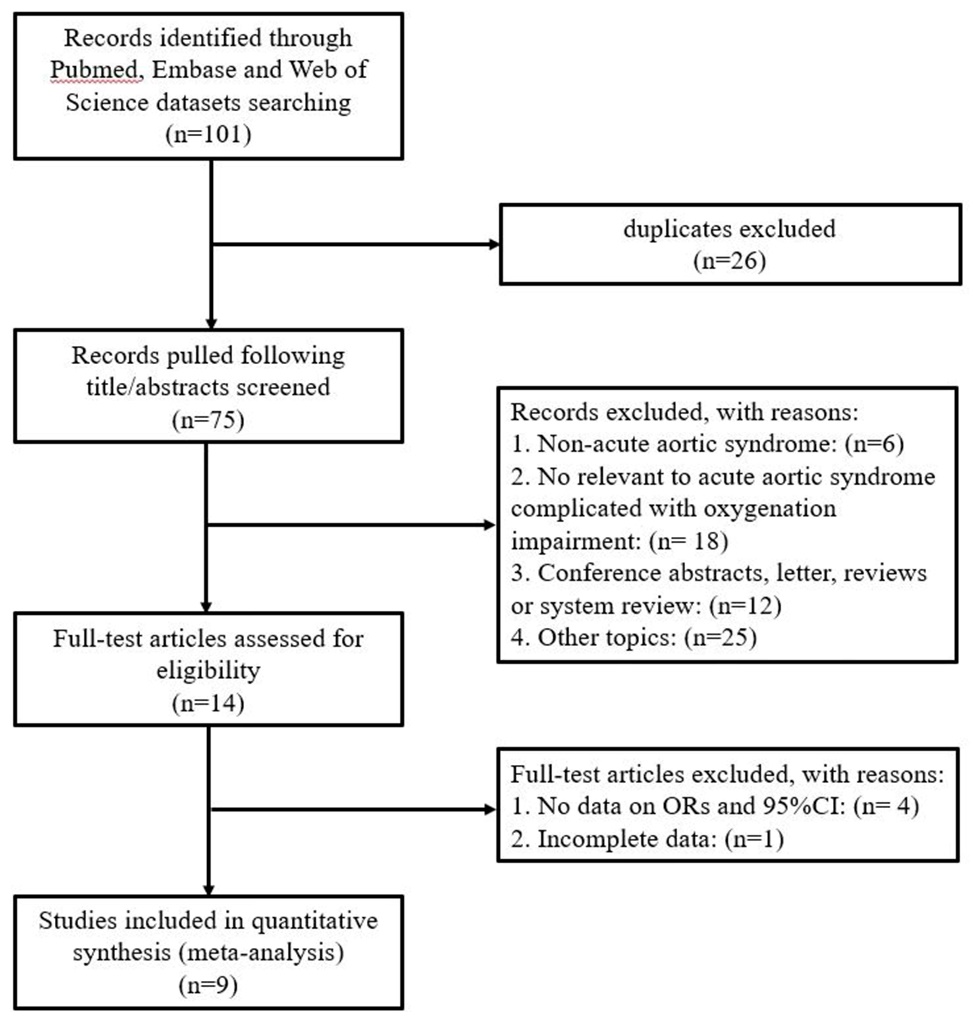


**Supplementary Figure 2.** Flow diagram of literatures screen with criteria.

**Supplementary Figure 3**


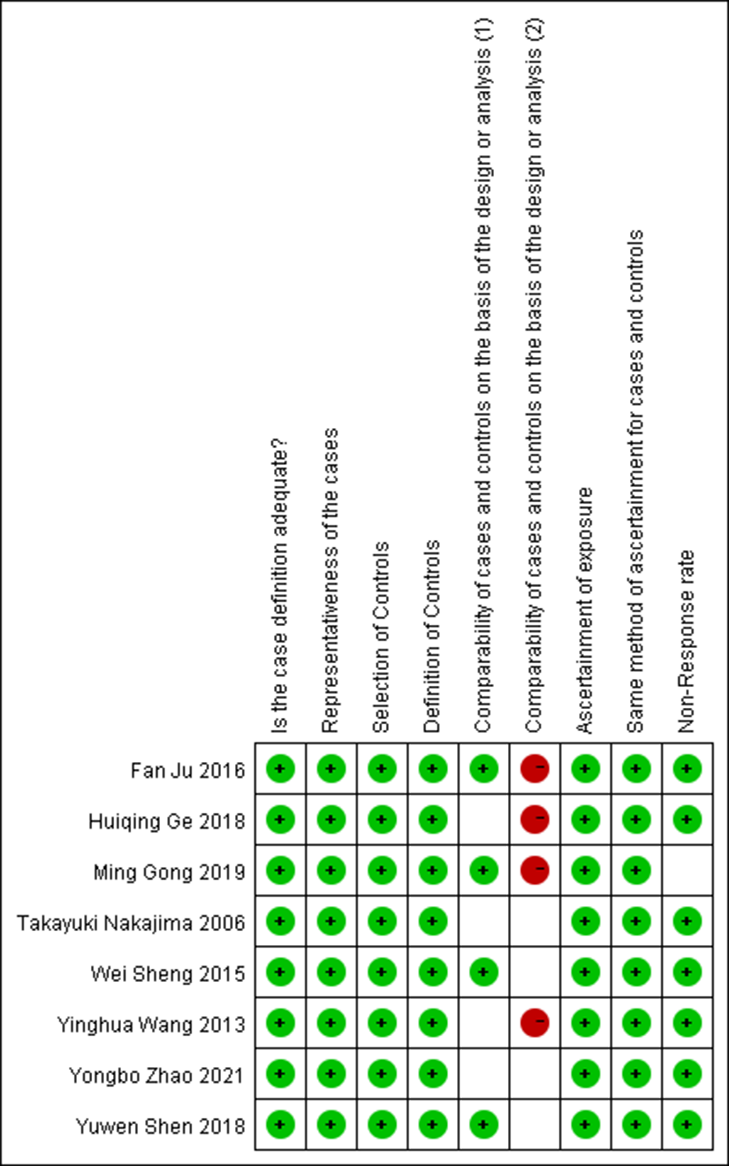


**Supplementary Figure 3.** Literature quality evaluation based on NOS assessment. NOS: Newcastle-Ottawa Scale.

**Supplementary Figure 4**


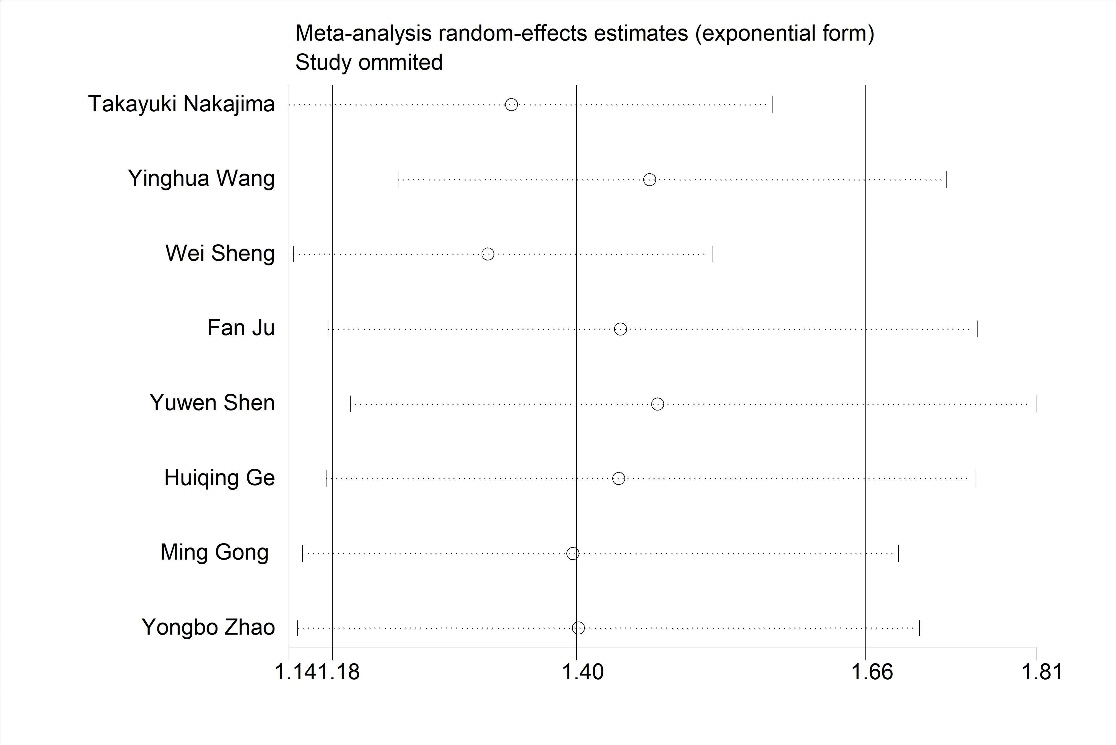


**Supplementary Figure 4.** The sensitivity analysis from the studies included.

**Supplementary Figure 5**


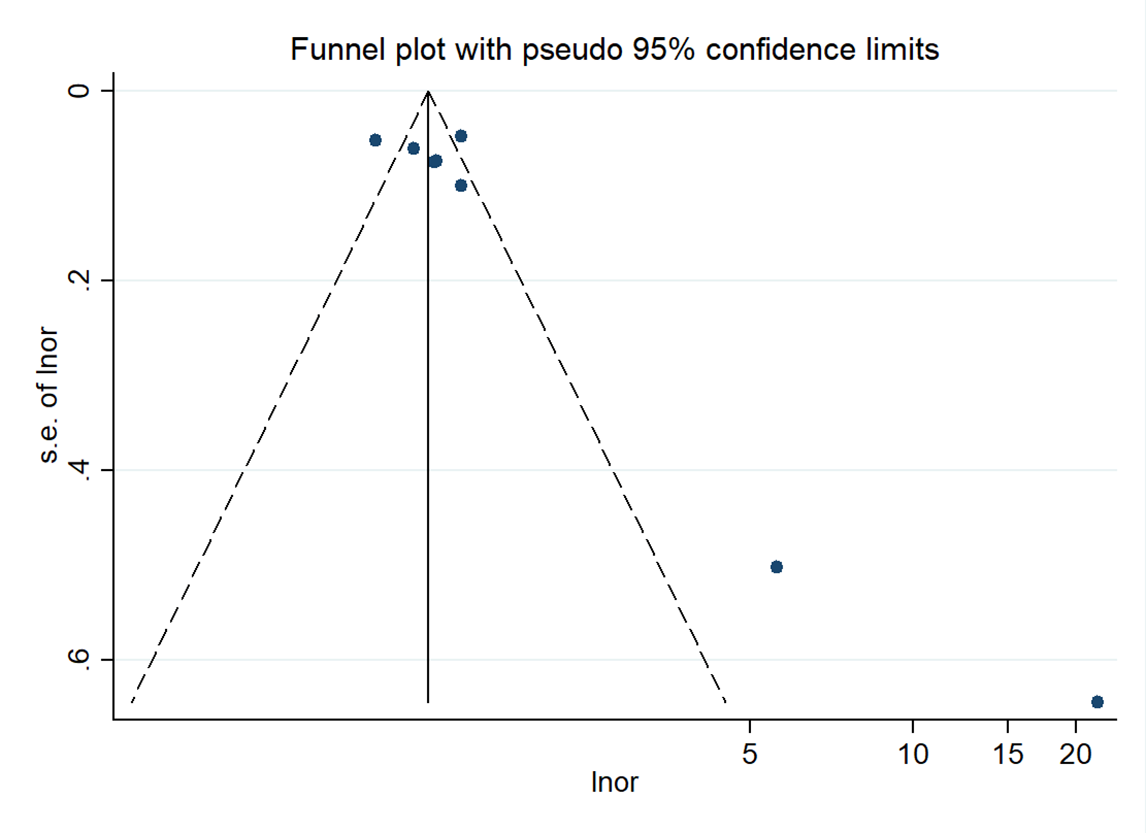


**Supplementary Figure 5.** Funnel plot of publication bias from the studies included.
