# Supplementary material for: Impact of body mass index on postoperative oxygenation impairment in patients with acute aortic syndrome
Source: Front Physiol. 2022 Aug 31;13:955702. doi: 10.3389/fphys.2022.955702 (PMC9470752; doi:10.3389/fphys.2022.955702)
Supplement: Supplementary file 2 [file Table2.DOCX]

**Supplementary Table 1.** Stratified analysis of preoperative clinical characteristics based on BMI.

|  | 18.5 ≤ BMI < 23  (n=73) | 23 ≤ BMI＜25  (n=42) | BMI ≥ 25  (n=112) | *P* values |
| --- | --- | --- | --- | --- |
| Age (years) | 54.00 (43.00-67.50) | 53.00 (44.25-57.25) | 52.00 (43.00-59.00) | 0.618 |
| Sex, male (%) | 49 (67.1) | 36 (85.7)^a^ | 92 (82.1)^c^ | 0.022 |
| BMI (kg/m^2^) | 21.33 (19.83-22.49) | 24.19 (23.46-24.50)^a^ | 27.33 (26.03-29.05)^bc^ | < 0.001 |
| Current smoking, n (%) | 31 (42.5) | 21 (50.0) | 66 (58.9) | 0.087 |
| Time from symptom onset to surgery (h) | 32.25 (13.81-68.63) | 19.33 (11.10-98.13) | 20.67 (9.06-44.01) | 0.206 |
| Medical history |  |  |  |  |
| Hypertension, n (%) | 35 (47.9) | 34 (81.0)^a^ | 91 (81.3)^c^ | 0.000 |
| CAD, n (%) | 6 (8.2) | 3 (7.1) | 11 (9.8) | 0.852 |
| Hyperlipemia, n (%) | 1 (1.4) | 0 (0.0) | 7 (6.3) | 0.083 |
| Diabetes, n (%) | 1 (1.4) | 2 (4.8) | 1 (0.9) | 0.254 |
| Fatty liver, n (%) | 6 (12.0) | 8 (22.2) | 50 (61.7)^bc^ | 0.000 |
| Marfan syndrome, n (%) | 2 (2.7) | 1 (2.4) | 0 (0.0) | 0.225 |
| COPD, n (%) | 2 (2.7) | 1 (2.4) | 1 (0.9) | 0.611 |
| Chronic liver disease, n (%) | 3 (4.1) | 3 (7.1) | 3 (2.7) | 0.448 |
| Chronic kidney disease, n (%) | 2 (2.7) | 3 (7.1) | 1 (0.9) | 0.098 |
| Preoperative oxygenation impairment, n (%) | 4 (5.5) | 6 (14.3) | 50 (44.6)^bc^ | < 0.001 |
| Vital signs on admission |  |  |  |  |
| Respiratory rate (/min) | 20.00 (20.00-20.00) | 20.00 (18.75-20.00) | 20.00 (18.00-20.00) | 0.822 |
| SBP (mmHg) | 137.00 (113.50-154.00) | 132.50 (120.00-155.50) | 142.00 (129.25-167.75) | 0.085 |
| DBP (mmHg) | 65.00 (61.00-76.00) | 68.00 (62.00-79.25) | 76.50 (65.00-84.75)^c^ | 0.001 |
| Heart rate (/min) | 76.64 ± 16.31 | 77.48 ± 16.28 | 81.44 ± 16.13 | 0.112 |
| Preoperative laboratory data |  |  |  |  |
| Hb (g/L) | 127.00 (114.00-138.00) | 129.00 (121.00-135.00) | 134.00 (122.00-145.00) | 0.052 |
| WBC (×10^9^/L) | 9.60 (7.05-13.15) | 10.15 (6.88-11.90) | 11.25 (8.33-14.80)^bc^ | 0.034 |
| PLT (×10^9^/L) | 165.00 (138.00-206.50) | 175.50 (126.50-229.00) | 182.50 (133.25-229.00) | 0.536 |
| D-dimer (μg/mL) | 1.45 (0.54-2.48) | 1.40 (0.50-3.30) | 1.52 (0.73-2.79) | 0.583 |
| FDP (mg/L) | 15.50 (6.00-34.4) | 13.95 (5.45-38.65) | 14.70 (7.40-26.70) | 0.983 |
| Cr (μmol/L) | 87.7 (69.10-105.25) | 96.40 (76.03-136.80) | 96.50 (80.93-111.80) | 0.057 |
| CRP (mg/L) | 21.80 (3.78-83.40) | 28.20 (9.06-124.50) | 53.40 (11.15-95.05) | 0.253 |
| Preoperative ultrasound and CT findings |  |  |  |  |
| Stanford classification |  |  |  | 0.631 |
| Type A, n (%) | 50 (68.5) | 27 (64.3) | 80 (72.1) |  |
| Type B, n (%) | 23 (31.5) | 15 (35.7) | 31 (27.9) |  |
| Aortic dissection, n (%) | 64 (91.4) | 39 (95.1) | 99 (92.5) | 0.769 |
| Intramural hematoma, n (%) | 11 (15.7) | 6 (14.6) | 18 (16.8) | 0.944 |
| LVEDD (mm) | 49.00 (45.00-54.00) | 51.00 (45.25-58.50) | 51.00 (47.00-54.00) | 0.324 |
| LVEF (%) | 59.00 (56.00-65.00) | 61.00 (56.00-68.75) | 60.00 (56.00-65.00) | 0.393 |
| Aortic regurgitation, n(%) | 64 (91.4) | 28 (77.8) | 84 (82.4) | 0.123 |
| Pleural effusion, n(%) | 24 (38.7) | 18 (52.9) | 37 (39.8) | 0.344 |
| Medications on admission |  |  |  |  |
| Vasodilators, n (%) | 47 (64.4) | 24 (57.1) | 82 (73.2) | 0.133 |

Data are presented as mean ± SD, n (%), or medians (interquartile ranges). BMI: body mass index; AAS: acute aortic syndrome; BMI: body mass index; CAD: coronary artery disease; COPD: chronic obstructive pulmonary disease; SBP: systolic blood pressure; DBP: diastolic blood pressure; WBC: white blood cell; Hb: hemoglobin; PLT: platelet; FDP: Fibrinogen degradation products; Cr: creatinine; CRP: C reactive protein; LVEDD: left ventricular end-diastolic dimension; LVEF: left ventricular ejection fraction.

^a^ *P* < 0.05 for 18.5 ≤ BMI < 23 group vs 23 ≤ BMI < 25 group;

^b^ *P* < 0.05 for 23 ≤ BMI < 25 group vs BMI ≥ 25 group;

^c^ *P* < 0.05 for 18.5 ≤ BMI < 23 group vs BMI ≥ 25 group.

**Supplementary Table 2.** Stratified analysis of intraoperative and postoperative clinical characteristics based on BMI.

|  | 18.5 ≤ BMI < 23  (n=73) | 23 ≤ BMI < 25  (n=42) | BMI ≥ 25  (n=112) | *P* values |
| --- | --- | --- | --- | --- |
| Intraoperative data |  |  |  |  |
| Aortic root replacement, n (%) | 43 (58.9) | 19 (45.2) | 72 (64.3) | 0.101 |
| Hemiarch replacement, n (%) | 10 (13.7) | 2 (4.8) | 7 (6.3) | 0.131 |
| Total arch replacement, n (%) | 37 (50.7) | 24 (57.1) | 67 (59.8) | 0.470 |
| Stented elephant trunk, n (%) | 34 (46.6) | 26 (61.9) | 62 (55.4) | 0.253 |
| Bentall operation, n (%) | 17 (23.3) | 9 (21.4)^a^ | 11 (9.8)^bc^ | 0.032 |
| David operation, n (%) | 1 (1.4) | 0 (0.0) | 1 (0.9) | 0.751 |
| TEVAR, n (%) | 21 (28.8) | 8 (19.0) | 31 (27.7) | 0.479 |
| CABG operation, n (%) | 7 (9.6) | 1 (2.4)^a^ | 1 (0.9)^bc^ | 0.010 |
| CPB time (h) | 3.38 (2.87-4.91) | 3.32 (2.73-3.88) | 3.45 (2.79-4.38) | 0.480 |
| Cross-clamp time (h) | 1.65 (1.35-2.49) | 1.47 (1.13-2.18) | 1.53 (1.28-2.06) | 0.176 |
| Circulatory arrest time (min) | 29.50 (20.25-35.75) | 26.50 (18.00-41.50) | 30.50 (22.75-41.00) | 0.440 |
| Operation time (h) | 6.29 (4.31-7.65) | 6.83 (4.17-7.71) | 6.64 (4.79-8.17) | 0.669 |
| Nasopharyngeal temperature (℃) | 25.00 (23.63-26.50) | 25.30 (24.33-26.75) | 25.00 (24.50-27.00) | 0.855 |
| RBC transfusion (u) | 3.50 (2.00-5.50) | 4.00 (2.00-6.00) | 4.00 (2.00-6.00) | 0.684 |
| PLT transfusion (u) | 1.00 (1.00-2.00) | 1.50 (1.00-2.00) | 1.00 (1.00-2.00) | 0.179 |
| Plasma transfusion (mL) | 460.00 (320.00-600.00) | 400.00 (350.00-600.00) | 600.00 (400.00-700.00) | 0.100 |
| Postoperative data |  |  |  |  |
| CRP (mg/L) | 120.58 (51.70-165.00) | 141.00 (78.50-207.00) | 135.50 (86.43-207.75) | 0.455 |
| Mechanical ventilation time (h) | 40.26 (18.11-89.31) | 43.08 (24.72-131.68) | 64.53 (29.35-138.38)^c^ | 0.039 |
| Tracheotomy, n (%) | 72 (98.6) | 41 (97.6) | 109 (97.3) | 0.836 |
| Postoperative oxygenation impairment, n (%) | 20 (27.4) | 19 (45.2)^a^ | 70 (62.5)^bc^ | < 0.001 |
| Pneumonia, n (%) | 43 (58.9) | 25 (59.5) | 83 (74.1) | 0.057 |
| Cerebral infarction, n (%) | 0 (0.0) | 3 (7.1) | 8 (7.1) | 0.065 |
| Delirium, n (%) | 25 (34.2) | 15 (35.7) | 43 (38.4) | 0.842 |
| AKI, n (%) | 7 (9.6) | 5 (11.9) | 16 (14.3) | 0.634 |
| Renal replacement therapy, n (%) | 2 (2.7) | 5 (11.9) | 7 (6.3) | 0.144 |
| ICU stay (days) | 5.00 (4.00-8.00) | 6.00 (4.00-10.00) | 6.00 (4.00-8.00) | 0.458 |
| Hospital stay (days) | 11.00 (9.00-14.50) | 13.50 (9.75-18.75) | 13.00 (10.00-18.00) | 0.075 |
| In hospital death, n (%) | 8 (11.0) | 6 (14.3) | 5 (4.5) | 0.092 |

Data are presented as mean ± SD, n (%), or medians (interquartile ranges). BMI: body mass index; AAS: acute aortic syndrome; TEVAR: thoracic endovascular aortic repair; CABG: coronary artery bypass grafting; CPB: cardiopulmonary bypass; RBC: red blood cell; PLT: platelet; CRP: C reactive protein; AKI: acute kidney injury; ICU: intensive care unit.

^a^ *P* < 0.05 for 18.5 ≤ BMI < 23 group vs 23 ≤ BMI < 25 group;

^b^ *P* < 0.05 for 23 ≤ BMI < 25 group vs BMI ≥ 25 group;

^c^ *P* < 0.05 for 18.5 ≤ BMI < 23 group vs BMI ≥ 25 group.

**Supplementary Table 3.** Characteristic of the studies included.

| First author | Year | Country | Ethnicity | Study size, sex (M/F), number of cases | Diagnostic criteria of oxygenation impairment | BMI | OR  (95% CI) | Adjustment factors | NOS score |
| --- | --- | --- | --- | --- | --- | --- | --- | --- | --- |
| Takayuki Nakajima | 2006 | Japan | Asian | 114, (60/54), 58 | P/F ratio ≤ 200 | Categorical  ≤ 25  > 25 | 1.00  5.62 (2.10-15.01) | Preoperative P/F, operation time, red cell concentrates | 7 |
| Yinghua Wang | 2013 | China | Asian | 186, (100/86), 92 | P/F ratio ≤ 200 | Continuous | 1.02 (0.92-1.13) | Preoperative hypoxemia, acute onset, CA time, postoperative transfusion ≥ 3000ml | 7 |
| Wei Sheng | 2015 | China | Asian | 192, (152/40), 55 | P/F ratio ≤ 200 | Categorical  ≤ 25  > 25 | 1.00  21.93 (6.21-77.48) | Smoking history, time from onset to operation, preoperative P/F, DHCA, blood transfusion in 24 hours postoperatively, mechanical ventilation time, ICU stay, length of stay | 8 |
| Fan Ju | 2016 | China | Asian | 403, (287/116), 69 | P/F ratio ≤ 100 | Continuous | 1.31 (1.13-1.52) | Age, preoperative Mb, ALT and Scr level, CPB time, reoperation within 48 hours, | 8 |
| Yuwen Shen | 2018 | China | Asian | 169, (121/48), 82 | P/F ratio ≤ 200 | Continuous | 1.20 (1.07-1.36) | Preoperative oxygenation impairment, preoperative WBC and Scr level, CPB time, CA time, last intraoperative central venous pressure, packed red blood cell transfusion, plasma transfusion, postoperative Scr, postoperative nervous system dysfuncion | 8 |
| Huiqing Ge | 2018 | China | Asian | 211, (151/60), 43 | P/F ratio < 200 | Continuous | 1.32 (1.15-1.54) | Age, preoperative P/F, Stanford classification, WBC; HCT, CPB time, pH | 7 |
| Ming Gong | 2019 | China | Asian | 112, (83/29), 41 | P/F ratio ≤ 100 | Continuous | 1.47 (1.21-1.79) | Gender, WBC count, the duration of operation, CPB time, nasopharyngeal temperature, intraoperative amount of plasma, reoperation for bleeding, postoperative dialysis, cerebral infarction or bleeding, diabetes mellitus | 7 |
| Yongbo Zhao | 2021 | China | Asian | 64, (41/23), 36 | P/F ratio < 200 | Categorical  < 25  ≥ 25 | 1.00  1.47 (1.15-1.39) | Time from onset to operation, preoperative WBC, hemoglobin, ALT, Scr and P/F level, intraoperative blood transfusions, assisted mechanical ventilation time, ICU stay | 7 |

M/F: male/female; BMI: body mass index; NOS: Newcastle-Ottawa Scale; P/F: arterial oxygen tension / inspiratory oxygen fraction; DHCA: deep hypothermic circulatory arrest; ICU: intensive care unit; CA: circulatory arrest; Mb: myoglobin; ALT: alanine transaminase; Scr: serum creatinine; CPB: cardiopulmonary bypass; WBC: white blood cell; HCT: hematocrit.

**Supplementary Table 4.** Results of subgroup analysis of the studies included.

|  |  | Fixed-effect model | |  | Random-effect model | |  | Heterogeneity | |
| --- | --- | --- | --- | --- | --- | --- | --- | --- | --- |
|  | N | OR (95%Cl) | *P* |  | OR (95%Cl) | *P* |  | *I*^2^% | *Q* |
| All study | 8 | 1.28 (1.21-1.34) | < 0.001 |  | 1.40 (1.18-1.66) | < 0.001 |  | 88.0 | 0.000 |
| Diagnostic criteria of oxygenation impairment |  |  |  |  |  |  |  |  |  |
| P/F ≤ 100 | 2 | 1.26 (1.19-1.33) | < 0.001 |  | 1.44 (1.15-1.82) | < 0.001 |  | 91.1 | < 0.001 |
| P/F ≤ 200 | 6 | 1.37 (1.21-1.54) | < 0.001 |  | 1.37 (1.21-1.54) | 0.002 |  | 0.0 | 0.358 |
| Age group |  |  |  |  |  |  |  |  |  |
| ≤ 50 years | 3 | 1.35 (1.23-1.48) | < 0.001 |  | 1.35 (1.23-1.48) | < 0.001 |  | 0.0 | 0.614 |
| > 50 years | 4 | 1.24 (1.17-1.32) | < 0.001 |  | 1.38 (1.04-1.83) | 0.025 |  | 93.5 | < 0.001 |
| Sample size |  |  |  |  |  |  |  |  |  |
| ≤ 200 | 6 | 1.26 (1.19-1.34) | < 0.001 |  | 1.50 (1.18-1.92) | 0.001 |  | 91.4 | < 0.001 |
| > 200 | 2 | 1.32 (1.19-1.46) | < 0.001 |  | 1.32 (1.19-1.46) | < 0.001 |  | 0.0 | 0.943 |
| Different forms of BMI |  |  |  |  |  |  |  |  |  |
| categorical | 3 | 1.51 (1.38-1.66) | < 0.001 |  | 5.15 (1.05-25.36) | 0.044 |  | 91.8 | < 0.001 |
| continuous | 5 | 1.19 (1.12-1.27) | < 0.001 |  | 1.24 (1.09-1.40) | 0.001 |  | 76.0 | 0.002 |

OR: odds ratio; P/F: arterial oxygen tension / inspiratory oxygen fraction; BMI: body mass index.
